# Effect of Prophylactic Low Level Laser Therapy on Oral Mucositis: A Systematic Review and Meta-Analysis

**DOI:** 10.1371/journal.pone.0107418

**Published:** 2014-09-08

**Authors:** Sapna Oberoi, Gabriele Zamperlini–Netto, Joseph Beyene, Nathaniel S. Treister, Lillian Sung

**Affiliations:** 1 Division of Haematology/Oncology, The Hospital for Sick Children, Toronto, Ontario, Canada; 2 Program in Child Health Evaluative Sciences, The Hospital for Sick Children, Peter Gilgan Centre for Research and Learning, Toronto, Ontario, Canada; 3 Department of Clinical Epidemiology & Biostatistics, McMaster University, Hamilton, Ontario, Canada; 4 Division of Oral Medicine & Dentistry, Brigham and Women's Hospital, Boston, Massachusetts, United States of America; MGH, MMS, United States of America

## Abstract

**Background:**

Objective was to determine whether prophylactic low level laser therapy (LLLT) reduces the risk of severe mucositis as compared to placebo or no therapy.

**Methods:**

MEDLINE, EMBASE, and Cochrane Central Register of Controlled Trials were searched until February 2014 for randomized controlled trials (RCTs) comparing prophylactic LLLT with placebo or no therapy in patients with cancer or undergoing hematopoietic stem cell transplantation (HSCT). All analyses used random effects models.

**Results:**

Eighteen RCTs (1144 patients) were included. Prophylactic LLLT reduced the overall risk of severe mucositis (risk ratio (RR) 0.37, 95% confidence interval (CI) 0.20 to 0.67; *P* = 0.001). LLLT also reduced the following outcomes when compared to placebo/no therapy: severe mucositis at the time of anticipated maximal mucositis (RR 0.34, 95% CI 0.20 to 0.59), overall mean grade of mucositis (standardized mean difference −1.49, 95% CI −2.02 to −0.95), duration of severe mucositis (weighted mean difference −5.32, 95% CI −9.45 to −1.19) and incidence of severe pain (RR 0.26, 95% CI 0.18 to 0.37).

**Conclusion:**

Prophylactic LLLT reduced severe mucositis and pain in patients with cancer and HSCT recipients. Future research should identify the optimal characteristics of LLLT and determine feasibility in the clinical setting.

## Introduction

Oral mucositis is one of the most frequent and distressing complications observed in patients receiving cancer treatment [1]. Mucositis develops in approximately 20–40% of patients receiving conventional chemotherapy, 60–85% of patients undergoing hematopoietic stem cell transplantation (HSCT) and in nearly all patients with head and neck cancer receiving radiation [1]–[3]. Chemotherapy associated mucositis typically peaks at 7 to 14 days after the initiation of chemotherapy and resolves within a few days as compared to radiotherapy associated mucositis in head and neck cancer patients, which peaks at weeks 4 to 6 of treatment and usually lasts for weeks after completion of radiation [3]–[6]. Oral mucositis is associated with pain, infections, need for enteral or parental nutrition, impaired nutritional status and quality of life, increased duration and cost of hospital stay, and interruptions or dose reductions in chemotherapy or radiotherapy [1], [7]–[10]. Because of these implications, research has been focused on different preventive and treatment strategies for oral mucositis.

The Multinational Association of Supportive Care in Cancer (MASCC) and the International Society of Oral Oncology (ISOO) have recently published guidelines for the prevention of oral mucositis [11]. One recommended intervention was low level laser therapy (LLLT), also referred to as photobiomodulation, in patients receiving HSCT with or without total body irradiation (level of evidence II) and in patients receiving head and neck radiotherapy without concomitant chemotherapy (level of evidence III) [11]. These recommendations were based on a systematic review which included studies published up to December 2010 that did not synthesize the data [12]. In contrast to the MASCC/ISOO recommendations and systematic review, a Cochrane Collaboration systematic review published in 2011 found that there was only weak evidence from two small studies at risk for bias favoring LLLT for the prevention of mucositis [13]. In their conclusions, LLLT was not one of the two interventions (cryotherapy and keratinocyte growth factor) found to have evidence of benefit [13]. The authors recommended that more randomized controlled trials (RCTs) are required for interventions such as laser therapy [13].

Since both of these systematic reviewers were conducted, there have been several RCTs performed evaluating the effect of prophylactic LLLT on oral mucositis [14]–[21]. Synthesis of all the evidence with careful evaluation of the risk of bias would permit a better evaluation of the effect of LLLT and may also provide insight into factors which may explain heterogeneity of the effect of the intervention.

Our primary objective was to determine whether prophylactic LLLT reduces the overall risk of severe mucositis in children and adults with cancer or undergoing HSCT as compared to placebo or no therapy. Our secondary objectives were to determine whether prophylactic LLLT reduces the incidence of severe mucositis when maximum mucositis is anticipated, overall mean mucositis grade, duration of severe mucositis, incidence of any pain and severe pain, overall mean pain scores, proportion of patients requiring opioid analgesia and unplanned radiotherapy interruption, as compared to placebo or no therapy.

## Materials and Methods

### Data Sources and Searches

We developed a protocol for this review and followed the Preferred Reporting Items for Systematic Reviews and Meta-analyses (PRISMA) statement [22]. Comprehensive searches for relevant trials using the Ovid platform in MEDLINE (from 1946 to February 17, 2014), EMBASE (from 1947 to February 17, 2014), Cochrane Central Register of Controlled Trials (to January2014), CINAHL (1983 to February 17, 2014), Web of Science (to February 17, 2014), SCOPUS (to February 2014), and LILACS (to February 2014) were performed without any language or publication status restriction. The search strategy included the following Medical Subject Heading terms: “mucositis”, “laser therapy”, “low-level laser therapy”, “phototherapy”, “light emitting diode”, “transplantation”, “chemotherapy” and “chemoradiotherapy”. Multiple synonyms, abbreviations, and related keywords for each of these terms were used for searching the databases. The search strategy is available as Appendix S1.

We also reviewed the conference proceedings of the International Society of Paediatric Oncology, American Society of Clinical Oncology, American Society of Hematology, American Society of Pediatric Hematology and Oncology, and Multinational Association of Supportive Care in Cancer from 2011 to 2013 to identify more recently completed studies. The reference lists of identified studies were also searched to identify further eligible studies.

### Study Selection

We defined inclusion and exclusion criteria *a priori*. We included RCTs and quasi-RCTs in this review. Case–control studies, cohort studies, case reports, case series, animal studies, letters to editors, editorials, review articles and commentaries were excluded. Studies were included if the population consisted of patients with cancer or undergoing HSCT and patients were randomly assigned to receive prophylactic LLLT versus placebo, no therapy or usual care. Studies were excluded if: (1) allocation not randomly assigned; (2) absence of a placebo or no treatment group; (3) randomized chemotherapy cycles or left and right buccal mucosa within a patient rather than randomizing patients (as episodes would not be independent); and (4) duplicate publication. Studies included in the meta-analysis were not restricted by language or publication status.

Two reviewers (SO and GZ) independently evaluated the titles and abstracts of publications identified by the search strategy. Any publication considered potentially relevant by either reviewer was retrieved in full and assessed for eligibility. Inclusion of studies in this meta-analysis was determined by agreement of both reviewers. Discrepancies were resolved by a third reviewer (LS). Agreement of study inclusion between reviewers was evaluated using the kappa statistic. Strength of agreement was defined as slight (0.00 to 0.20), fair (0.21 to 0.40), moderate (0.41 to 0.60), substantial (0.61 to 0.80), or almost perfect (0.81 to 1.00) [23].

### Type of Intervention

Interventions were internally (intraoral) or externally (extraoral) delivered LLLT given as prophylaxis for oral mucositis in any intensity, power, wavelength, energy density or schedule.

### Outcomes

The primary outcome was the overall incidence of severe mucositis over the entire observation period. Severe mucositis was defined as grades 3 or 4 mucositis on a 5 point grading scale ranging from 0 to 4. Three instruments were graded in this fashion, namely the World Health Organization (WHO) scale, the Radiation Therapy Oncology Group (RTOG) scale, and the National Cancer Institute Common Terminology Criteria (NCI CTC) (version 2) [24]–[26]. NCI CTCAE (version 3 and 4) uses a grading scale which ranges from 1 to 5; for our purpose, severe mucositis was considered grades 3 to 5 [27], [28]. We also included the Tardieu mucositis scale which ranges from grades 0 to 3 [29]. Grades 2 and 3 on the Tardieu scale are similar to grades 3 and 4 according to the other mucositis grading scales and consequently, we classified Tardieu scale scores of grade 2 and 3 as severe mucositis. However, we conducted a sensitivity analysis excluding studies using the Tardieu scale to evaluate the robustness of the results. In studies reporting severe mucositis by more than one of these 5 point grading scales, the WHO scale was used for primary outcome analysis if available.

The secondary outcomes were: (1) incidence of severe mucositis (defined using the same approach as the primary outcome) at the time point when maximum mucositis was expected, namely at week 6±1 of radiotherapy or chemo-radiotherapy in head and neck cancer patients and at day 10±4 of chemotherapy or HSCT (from the days of chemotherapy initiation or stem cell infusion respectively); (2) overall mean mucositis grade or score over the observation period as measured by any mucositis grading scale including continuous scales such as the Oral Mucositis Assessment Scale (OMAS) [30]; and (3) duration of severe mucositis (defined using the same approach as the primary outcome). We also evaluated oral pain for studies that used an 11 point pain visual analogue scale (VAS) ranging from 0 to 10 [31]. We examined the incidence of any pain defined as a pain score more than 0; incidence of severe pain defined as a pain score more than 7 [32]; and overall mean pain score. Other outcomes were the proportion of patients requiring opioid analgesia and proportion of patients with unplanned radiotherapy interruption due to mucositis in head and neck cancer patients.

### Risk of Bias Assessment

We used the Cochrane Collaboration's tool for assessing the risk of bias in randomized trials [33]. This tool includes the following domains relevant to internal validity: selection bias, performance bias, detection bias, attrition bias and reporting bias. We evaluated the following sources of bias related to these domains: random number generation, allocation concealment, blinding of participants and personnel, blinding of outcome assessment, incomplete outcome data and selective outcome reporting. We *a priori* prioritized allocation concealment and blinding for stratified analyses [34]. For blinding, we evaluated whether participant, personnel and outcome assessors were blinded versus studies in which at least one of these groups was not blinded.

### Data Extraction

A data abstraction form was developed by the authors and all information was abstracted in duplicate by two authors (SO and GZ). Where information was missing from a publication, the corresponding author was contacted and the missing information was requested.

### Data Synthesis

We combined data at the study level for this meta-analysis. For dichotomous outcomes such as the overall incidence of severe mucositis, data were synthesized using the risk ratio (RR) as the effect measure with its 95% confidence interval (CI). Risk ratios less than 1 suggest that LLLT is better than placebo or no therapy in preventing oral mucositis. Number needed to treat was calculated as the inverse of the absolute risk difference between groups. For continuous outcomes with missing summary measures, we made the following assumptions to facilitate data synthesis: the mean can be approximated by the median; the range contains six standard deviations (SDs), the 95% CI contains four standard errors (SEs), and the interquartile range contains 1.35 SDs. Where continuous outcomes were measured using different scales (such as the mean mucositis grade), outcomes were synthesized using the standardized mean difference (SMD). Where continuous outcomes were measured on the same scale (such as the pain VAS), outcomes were synthesized using the weighted mean difference (WMD). A SMD or WMD less than 0 indicate that the mean mucositis or pain VAS scores were lower in the LLLT arm as compared to the placebo or no therapy arm. Effect sizes of dichotomous and continuous outcomes were weighted by the Mantel-Haenzel and inverse variance methods respectively. As we anticipated heterogeneity between studies, a random effects model was used for all analyses. Statistical heterogeneity between trials was assessed using the I^2^ value, which describes the percentage of total variation across studies due to heterogeneity rather than chance [33].

Potential publication bias was explored by visual inspection of funnel plots when at least 10 studies were available [33]. Publication bias occurs when small studies are published only if the results are positive. A funnel plot is a graph with the effect (RR, SMD or WMD in our analysis) on the x-axis, and the inverse of variance of the effect on the y-axis. Asymmetry, without studies in the bottom right corner, suggests publication bias. In the event of potential publication bias, we used the “trim and fill” technique to determine the impact of such potential bias [33]. With this technique, outlying studies are deleted, and hypothetical negative studies with equal weight are created.

In order to explore sources of heterogeneity, stratified analyses were planned *a priori* for the primary outcome only (to limit the number of analyses performed). Factors evaluated were: (1) population age (adult versus pediatric (age ≤18 years)/combined adult and pediatric); (2) underlying condition (head and neck cancer patients receiving radiotherapy or chemo-radiotherapy versus chemotherapy or HSCT); (3) intraoral versus extraoral laser delivery; (4) energy density of laser (≤4 J/cm^2^ versus >4 J/cm^2^); (5) blinding of patients, providers and assessors (yes versus no or unclear); and (6) adequate allocation concealment (yes versus no or unclear).

Meta-analyses were conducted using Review Manager 5.2 (Cochrane Collaboration, Nordic Cochrane Centre).

## Results

**Figure 1:**
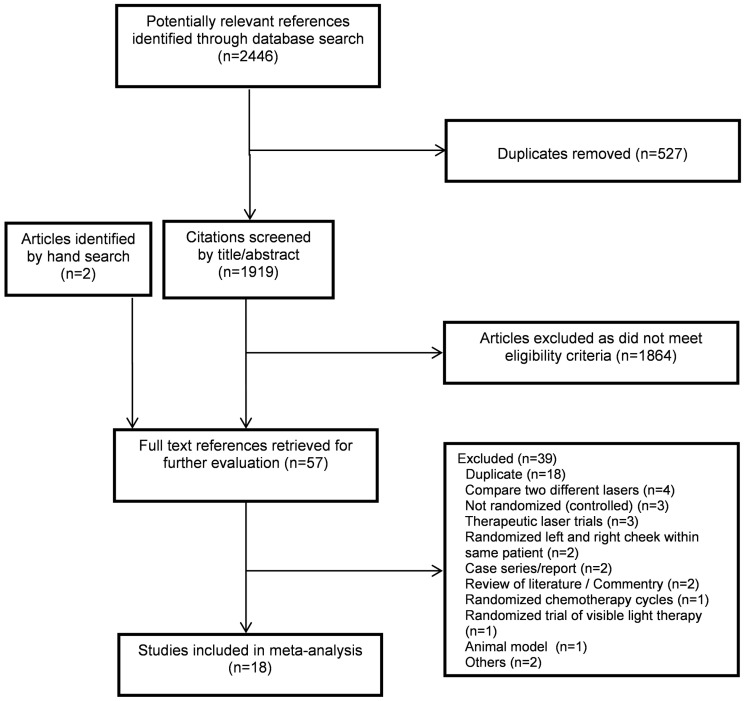
Flow diagram of trial identification and selection.

The flow diagram of trial identification, selection and reasons for exclusion is presented in Figure 1. A total of 2445 citations were identified by the search strategy; 18 studies met the eligibility criteria and were included in this systematic review [14]–[21], [35]–[48]. Agreement between reviewers regarding study inclusion was almost perfect (kappa 0.89, 95% CI 0.78 to 1.00). All studies except one were published as full text articles [38]. One study reported outcomes as a stratified analysis by underlying disease diagnosis and HSCT regimen [19] and thus, represented two separate analyses for a total of 19 prophylactic LLLT comparisons randomizing 1144 patients.

Table 1 lists the baseline characteristics of the studies, patient population, intervention and mucositis evaluation schedule and scale. The earliest trial was published in 1997 with 9 (47%) studies being published in 2012 and 2013. Half of the trials were from Brazil [14], [18], [20], [38]–[41], [43], [44], [47]. Eight trials were conducted in the HSCT population, 8 in head and neck cancer patients receiving radiotherapy or chemo-radiotherapy and the remainder in other patients receiving chemotherapy. One study was a solely pediatric trial [41]. Intraoral laser therapy was used in all except two trials [19]. With respect to laser source, InGaAlP (6 trials) and helium-neon (5 trials) were the most commonly used lasers. The mean wavelength and energy density of the lasers used across the trials was 660±34.7 nm and 3.3±1.3 J/cm^2^.

**Table 1:** Baseline characteristics of studies included in the meta-analysis*.

First Author (reference)	Year Pub	Country	Age	Underlying Condition	Setting	No. Rando-mized	Type of Laser	Wave-length (nm)	Power Output (mW)	Irradiation Time per Spot (sec)	Energy per Spot (Joules)	Energy Density (J/cm2)	Laser Schedule	Oral Mucositis Evaluation Schedule	Mucositis Assessment Scale
Antunes [14], [36]	2013	Brazil	Adults	Head and neck cancer	Chemo-radio	94	InGaAIP	660	100	10	1	4	5 sessions/week during radiation	Daily	WHO and OMAS
Arbabi-Kalati [15]	2013	Iran	Adults	Oncologic disorders	Chemo	48	Mustang	630	30	NA	NA	5	Prior to chemotherapy	Two times/week	WHO
Gautam [16], [37]	2012	India	Adults	Head and neck cancer	Chemo-radio	239	He-Ne	632.8	24	125	3	3	5 sessions/week×45 days	Weekly	RTOG/EORTC
Gautam [17]	2012	India	Adults	Oral carcinoma	Chemo-radio	121	He-Ne	632.8	24	145	3.5	3.5	5 sessions/week during radiation	Weekly	RTOG/EORTC
Gouvea de Lima [18]	2012	Brazil	Adults	Head and neck cancer	Chemo-radio	75	GaAlAs	660	10	10	0.1	2.5	5 sessions/week during radiation	Every two weeks	NCI CTCv2
Hodgson (a) [19]	2012	USA	Both	Hematologic, oncologic disorders	HSCT (allo, auto)	40	Infrared LED	670±10	50	80	4	4	Daily from day 0 to day +14	Three times/week	WHO,NCI CTCAE and OMAS
Hodgson (b) [19]	2012	USA	Adults	Multiple myeloma	HSCT (auto)	40	Infrared LED	670±10	50	80	4	4	Daily from day 0 to day +14	Three times/week	WHO,NCI CTCAE and OMAS
Oton-Leite [20], [48]	2012	Brazil	Adults	Head and neck cancer	Radio or Chemo-radio	60	InGaAlP	685	35	25	0.8	2	5 sessions/week during radiation	Mid and at the end of treatment (week 3 and week 6)	WHO
Pires-Santos [38]	2012	Brazil	Adults	Breast cancer	Chemo	12	NA	NA	NA	NA	NA	NA	Day 0 to day +7 q 48 hours	NA	NA
Silva [21]	2011	Brazil	Both	Hematologic, oncologic disorders	HSCT (allo, auto)	42	InGaAIP	660	40	4	0.16	4	Daily from day −4 to day +4	Daily	WHO
Chor [39]	2010	Brazil	Adults	NA	HSCT (auto)	34	AsGaAl	660	50	NA	NA	NA	Daily from day −7 to day 0	Daily	Tardieu
Khouri [47]	2009	Brazil	Both	Hematologic disorders	HSCT (allo)	22	InGaAIP and GaAlAs	660 and 780	25	10	0.25	6.3	Daily until day +15 or day of engraftment	NA	WHO and OMAS
Antunes [40]	2007	Brazil	Adults	Hematologic Disorders	HSCT (allo, auto)	38	InGaAIP	660	46.7	16.7	0.8	4	Daily from day −7 until neutrophil recovery	Daily	WHO and OMAS
Cruz [41]	2007	Brazil	Children	Hematologic and solid malignancies	Chemo or HSCT (auto)	62	NA	780	60	NA	NA	4	Daily from start of chemo×5 days	Day +8 and day +15	NCI CTC
Schubert [42]	2007	USA	Both	Hematologic, oncologic disorders	HSCT (allo, auto)	47	GaAlAs	650	40	2	0.08	2	Daily from day −1 of conditioning to day +2	Two times/week	OMI [55]
Arun Maiya [35]	2006	India	Adults	Oral carcinoma	Radio	50	He-Ne	632.8	10	180	1.8	1.8	5 sessions/week during radiation	Once at the end of treatment (week 6)	WHO
Lopes [43]	2006	Brazil	Adults	Head and neck cancer	Chemo-radio	60	InGaAlP	685	35	58	2	2	NA	Pretreatment, 4 weeks and at the end of therapy	NCI CTC
Bensadoun [44], [45]	1999	France	Adults	Head and neck cancer	Radio	30	He-Ne	632.8	60	33	2	2	5 sessions/week during radiation	Weekly	WHO
Cowen [46]	1997	France	Adults	Hematologic malignancies	HSCT (auto)	30	He-Ne	632.8	60	10	0.6	1.5	Daily from day −5 to day −1	Daily	Tardieu

Abbreviations: Allo - allogeneic hematopoietic stem cell transplant; Auto-autologous hematopoietic stem cell transplant; Chemo – chemotherapy; EORTC-European Organization for Research and Treatment of Cancer; GaAIAs/AsGaAI – gallium-aluminium-arsenide/arsenate; He-Ne- helium-neon; HSCT – hematopoietic stem cell transplantation; InGaAIP – indium-gallium-aluminium phosphide; LED – light emitting diode; NA – not available; NCI CTC – National Cancer Institute Common Terminology Criteria; OMAS – Oral Mucositis Assessment Scale; OMI - Oral Mucositis Index; Pub – published; Radio- radiotherapy; RTOG – Radiation Therapy Oncology Group; VAS – visual analog scale; WHO – World Health Organization.

*There were 18 studies reporting 19 separate comparisons between low level light therapy and placebo/no therapy as one study stratified the population by underlying disease diagnosis and HSCT regimen.

Summary of the risk of bias of included studies is presented in Appendix S2. The number of studies at low risk of bias was as follows: for random sequence generation (n = 13, 68%), allocation concealment (n = 4, 21%), blinding of participants and personnel (n = 13, 68%), blinding of outcome assessor (n = 15, 79%), incomplete outcome data (n = 15, 79%) and selective outcome reporting (n = 13, 68%). There were four studies (21%) that were at low risk of bias across all domains.

### Primary Outcome

**Figure 2:**
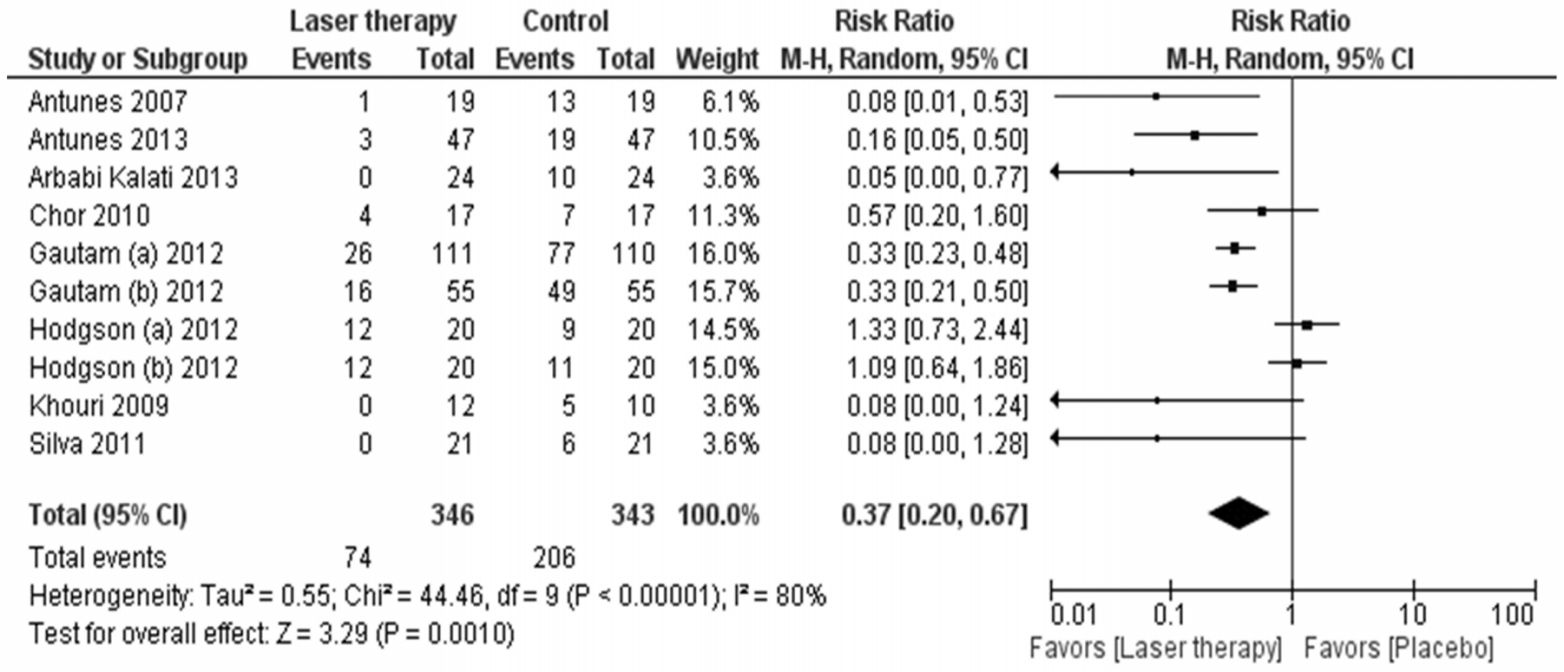
Forest plot of overall incidence of severe (grade 3 or 4) mucositis. Squares to the left of the vertical line indicate that low level laser therapy reduces mucositis. Horizontal lines through the squares represent 95% confidence intervals (CIs). The size of the squares reflects each study's relative weight, and the diamond represents the aggregate risk ratio and 95% CI.

Ten studies encompassing 689 patients reported the overall incidence of severe mucositis using the WHO (n = 7), RTOG (n = 2) and Tardieu (n = 1) scales. Prophylactic LLLT reduced the risk of severe mucositis when compared to placebo or no therapy (RR 0.37, 95% CI 0.20 to 0.67; *P* = 0.001; Table 2 and Figure 2). The sensitivity analysis excluding the single study using the Tardieu scale did not affect the estimate of LLLT treatment effect (RR 0.34, 95% CI 0.18 to 0.65; *P* = 0.001).The absolute risk reduction in the incidence of severe mucositis with LLLT was −0.35 (95% CI −0.48 to −0.21; *P*<0.0001), resulting in a number needed to treat of three patients to prevent one episode of severe mucositis.

**Table 2:** Summary of outcomes of low level laser therapy as compared to placebo/no treatment.

Outcome	Number Studies	Number Patients	Effect	95% CI¥	I^2^	*P*
Overall incidence of severe (grade 3 or 4) mucositis	10	689	RR 0.37	0.20 to 0.67	80%	0.001
Incidence of severe (grade 3 or 4) mucositis at anticipated time of maximal mucositis*	6	546	RR 0.34	0.20 to 0.59	62%	0.0001
Overall mean grade of mucositis	8	603	SMD −1.49	−2.02 to −0.95	86%	<0.0001
Duration of severe (grade 3 or 4) mucositis	3	361	WMD −5.32	−9.45 to −1.19	94%	0.01
Incidence of any pain	7	591	RR 0.89	0.76 to 1.04	96%	0.15
Incidence of severe pain**	2	331	RR 0.26	0.18 to 0.37	0%	<0.0001
Overall mean pain scores	5	222	WMD −2.46	−4.41 to −0.77	97%	0.004
Number of patients requiring opioid analgesia	5	530	RR 0.47	0.37 to 0.60	0%	<0.0001
Unplanned radiotherapy interruption due to mucositis in head and neck cancer patients	5	560	RR 0.23	0.12 to 0.44	0%	<0.0001

Abbreviations: RR - risk ratio; SMD - standardized mean difference; WMD – weighted mean difference; CI – confidence interval;

*Maximum anticipated mucositis was week 6±1 in head and neck cancer radiotherapy/chemo-radiotherapy trials and day 10±4 in chemotherapy and hematopoietic stem cell transplantation trials (from date of chemotherapy initiation and stem cell infusion respectively).

** Severe pain defined as a visual analogue scale score >7.

¥ All analyses used a random-effect model. A risk ratio <1 and a standardized mean difference or weighted mean difference <0 with 95% CIs that do not include 1 or 0 respectively, suggest that low level laser is better than placebo/no therapy.

### Secondary Outcomes

Table 2 summarizes the secondary outcomes of the analysis. Synthesis of six studies encompassing 546 patients showed a reduced risk of severe mucositis with LLLT at the time of anticipated maximal mucositis (RR 0.34, 95% CI 0.20 to 0.59; *P* = 0.0001) (Table 2 and Appendix S3). Prophylactic LLLT also reduced the overall mean grade of mucositis and duration of severe mucositis (Table 2).

Table 2 also illustrates that LLLT was associated with a reduction in the incidence of severe pain, overall mean pain scores, and the proportion of patients requiring opioid analgesia. Finally, LLLT reduced unplanned radiation interruption in head and neck cancer patients.

### Subgroup Analyses

Table 3 illustrates the stratified analyses for the primary outcome of severe mucositis. No interaction was seen between population age or underlying condition and the effect of LLLT. Studies using intraoral laser (RR 0.29, 95% CI 0.19 to 0.42) demonstrated a significantly larger reduction in severe mucositis compared to those using extraoral laser (RR 1.19, 95% CI 0.80 to 1.78; *P* for interaction <0.0001). There was a non-statistically significant larger effect of LLLT among studies utilizing >4 J/cm^2^ energy density as compared to ≤4 J/cm^2^ (*P* for interaction = 0.06). Studies with unclear or inadequate allocation concealment showed a larger treatment effect (*P* for interaction = 0.03).

**Table 3:** Effect of low level laser therapy as compared to placebo/no therapy on overall incidence of severe (grade 3 or 4) mucositis stratified by patient, laser and risk of bias characteristics.

Subgroup	Number Studies	Number patients	RR	95% CI¥	*P* for interaction
Population Age					0.90
Adult	8	607	0.33	0.18 to 0.59	
Pediatric or both adult/pediatric	2	82	0.41	0.02 to 10.87	
Underlying Condition					0.85
Chemotherapy or HSCT	7	264	0.35	0.13 to 0.98	
Head and neck cancer radiotherapy/chemo-radiotherapy	3	425	0.32	0.24 to 0.42	
Type of Laser Delivery					<0.0001
Intraoral	8	609	0.29	0.19 to 0.42	
Extraoral	2	80	1.19	0.80 to 1.78	
Energy Density of Laser					0.06
≤4 J/cm2	8	619	0.43	0.23 to 0.78	
>4 J/cm2	2	70	0.06	0.01 to 0.43	
Participants, Personnel and Assessors Blinded					0.11
Yes	8	625	0.42	0.23 to 0.76	
No or unclear	2	64	0.08	0.01 to 0.56	
Allocation Concealment Adequate					0.03
Yes	4	411	0.61	0.30 to 1.25	
No or unclear	6	278	0.16	0.07 to 0.41	

Abbreviations: RR – risk ratio; CI – confidence interval; HSCT – hematopoietic stem cell transplantation.

¥ All analyses used a random-effect model. A risk ratio <1 with 95% CIs that do not include 1, suggests that low level laser is better than placebo/no therapy.

### Other Analyses

**Figure 3:**
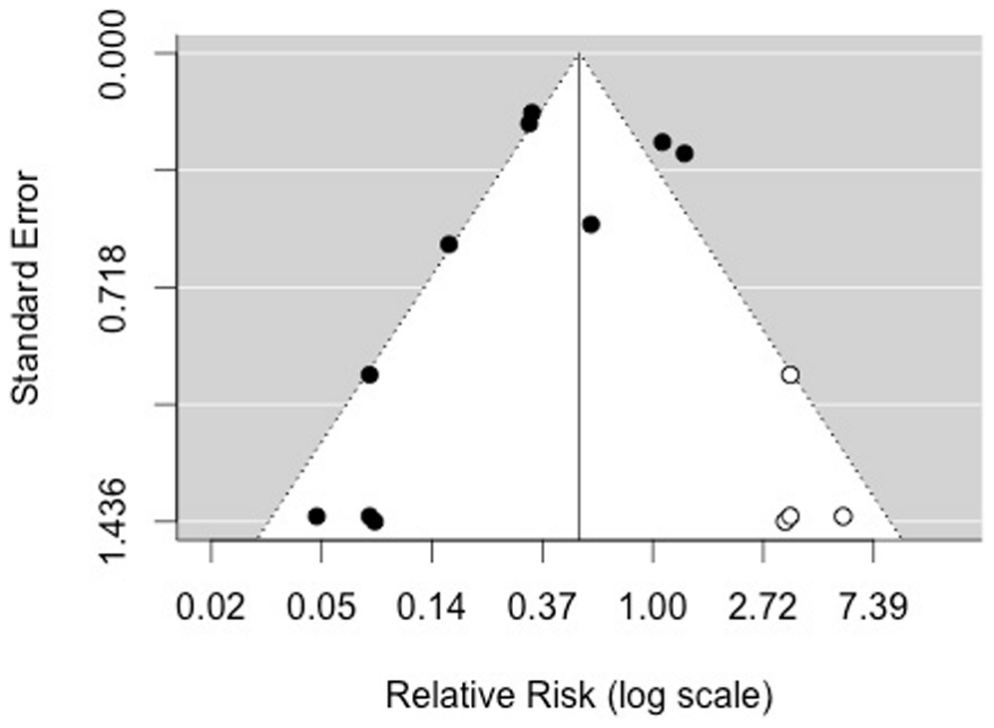
Funnel plot “trim and fill” technique assessing publication bias for overall incidence of severe mucositis. The x-axis represents the risk ratio for the effect of low level laser therapy and the y-axis represents the inverse of the variance of the effect. Estimated number of missing studies on right side = 4.

There were a sufficient number of studies for the primary outcome of severe mucositis to evaluate for publication bias. The funnel plot of risk of severe mucositis illustrated a potential for publication bias with an absence of studies in the right lower quadrant related to four studies (Figure 3) [15], [21], [40], [47]. When we used the “trim and fill” technique to account for this potential publication bias, the effect size of LLLT on severe mucositis was still statistically significant (RR 0.51, 95% CI 0.29 to 0.90; *P* = 0.0197). Funnel plots were not assessed for secondary outcomes because there were too few studies to permit these evaluations.

## Discussion

Our meta-analysis demonstrated that prophylactic LLLT reduces the overall risk of severe mucositis and other measures of mucositis severity including the duration of severe mucositis in patients with cancer and in those undergoing HSCT. Low level laser therapy also reduced the risk of severe pain, overall mean pain scores, need for opioid analgesia and unplanned radiotherapy interruptions. The consistency of the effect among the primary and secondary outcome measures strengthens the confidence in these results.

In general, the risk of bias scores were favorable with 79% of studies blinding the outcome assessor and 68% of studies reporting adequate random sequence generation. However, only 21% of studies reported adequate allocation concealment. This finding is important as lack of allocation concealment has been associated with exaggerated treatment effects [34]. However, even in studies that reported adequate allocation concealment, the treatment effect of RR 0.61 likely represents a clinically meaningful benefit of LLLT. A second issue is the potential for publication bias. However, we demonstrated that even with the addition of hypothetical negative studies of equal weight, the effect was still statistically significantly with a RR 0.51. When put together, these issues highlight that LLLT is likely effective in the prevention of oral mucositis although reported treatment effects may be exaggerated.

The findings of our meta-analysis provide unique and clinically important information in comparison to three prior systematic reviews with conflicting conclusions about the effect of LLLT [12], [13], [49]. All three prior reviews included fewer randomized prophylactic LLLT trials for a variety of reasons including searching fewer databases, date of last update, and use of restrictive eligibility criteria [12], [13], [49]. Two reviews included 5 and 8 prophylactic LLLT studies. The third review included both prophylactic and therapeutic randomized and nonrandomized LLLT studies and did not attempt to synthesize data [12], [50]. The Cochrane Collaboration systematic review used robust methodology. However, it only included 5 studies and limited their outcome to the mucositis evaluation on day 28 of therapy, which may not be appropriate when combining head and neck radiotherapy or chemo-radiotherapy trials with other chemotherapy and HSCT trials [13].

In our stratified analysis, we found a statistically significant and qualitative interaction by extraoral versus intraoral LLLT administration. This effect is biologically plausible if there is inadequate delivery of dose to the target tissues due to absorption of power by more superficial non-target tissues [19], [51]–[54]. However, this result should be considered hypothesis generating as there are other differences in trial design which may explain these results. For example, the two extraoral laser trials used non-coherent light emitting diodes and initiated LLLT later in comparison to the other trials [21], [39], [42], [46]. Additionally, confidence in this analysis is limited since there were only two studies in the extraoral LLLT group.

The major strengths of our meta-analysis included rigorous methodology for identification of studies and synthesis of data. The primary outcome for our review was the overall incidence of severe mucositis throughout the observation period, a clinically relevant outcome. However, similar to many systematic reviews, our analysis was limited by the methodological quality and outcome reporting of the included studies. Only four studies were at low risk of bias across all six domains used to evaluate validity. The studies were relatively heterogeneous with respect to laser parameters, laser schedules, mucositis assessment scales, time point of assessments and outcome reporting. Finally, only one study was conducted exclusively in children, limiting generalizability to the pediatric population.

A major question that remains to be answered is the feasibility of intraoral LLLT for use in routine clinical practice. The administration of this intervention requires the utilization of specialized equipment, trained personnel, involvement of a multi-disciplinary team and co-operation of patients as manipulation of the oral cavity may be painful during mucositis. Little is known about whether this intervention can be implemented in most settings and further, whether the intervention demonstrates effectiveness in routine clinical practice.

In conclusion, prophylactic LLLT reduced severe mucositis and pain in patients with cancer and HSCT recipients. Future research should identify the optimal characteristics of LLLT and determine feasibility in the clinical setting.

## Supporting Information

Appendix S1 **Search Strategies.** Search strategies used in MEDLINE, EMBASE and EBM. Other database strategies are available on request.
(DOC)

Appendix S2 Risk of bias assessment for included studies*.
(DOC)

Appendix S3 **Forest plot of incidence of severe (grade 3 or 4) mucositis at week 6±1 in head and neck cancer radiotherapy trials and at day 10±4 in chemotherapy or hematopoietic stem cell transplantation trials.** Squares to the left of the vertical line indicate that low level laser therapy reduces mucositis. Horizontal lines through the squares represent confidence intervals (CIs). The size of the squares reflects each study's relative weight, and the diamond represents the aggregate risk ratio and 95% CI.
(TIF)
