# Supplementary material for: Effect of Prophylactic Low Level Laser Therapy on Oral Mucositis: A Systematic Review and Meta-Analysis
Source: PLoS One. 2014 Sep 8;9(9):e107418. doi: 10.1371/journal.pone.0107418 (PMC4157876; doi:10.1371/journal.pone.0107418)
Supplement: Appendix S1 — Search Strategies. Search strategies used in MEDLINE, EMBASE and EBM. Other database strategies are available on request. (DOC) [file pone.0107418.s001.doc]

**Supplemental Appendix 1. Search Strategies**

Search strategies used in MEDLINE, EMBASE and EBM. Other database strategies are available on request.

**A) MEDLINE**

| Set | History | Comments |
| --- | --- | --- |
| 1 | mucositis/ or stomatitis/ or stomatitis, aphthous/ or stomatitis, herpetic/ or (stomatitis or (oral adj5 mucositis) or (aphthous adj5 (stomatitides or ulcer*)) or (canker adj5 sore*) or aphthae or (simplex adj2 oral adj2 herpes) or ((herpet* adj5 gingivostomatitides) or gingivostomatitis) or (herpetic adj5 stomatitides)).ti,ab. or Mouth Mucosa/ or ((oral or mouth) adj5 mucosa).ti,ab. | Oral mucositisterms |
| 2 | laser therapy/ or laser therapy, low-level/ or phototherapy/ or lasers/tu or ((laser* adj5 low adj5 (level* or power)) or "low-power laser*" or "low-level laser*" or (laser* adj5 (phototherap* or therap* or treatment* or treated or treating)) or lllt).ti,ab. or (lll or lle or "low-laser energy" or (low adj5 laser adj5 energy) or "light-emitting diode*" or (light adj5 emit* adj5 diode*) or LED or LEDs).ti,ab. | Laser therapy terms |
| 3 | exp neoplasms/ or exp Antineoplastic Agents/ or organ transplantation/ or exp tissue transplantation/ or transplantation, autologous/ or transplantation, heterologous/ or transplantation, heterotopic/ or exp transplantation, homologous/ or (neoplasm* or neoplas* or cancer* or oncolog* or tumor* or tumour* or transplant*).mp. or radiation dosage/ or dose-response relationship, radiation/ or Radiometry/ or Radiotherapy Dosage/ or (((gray or sievert) adj2 unit*) or (radiation adj2 (dosage* or dose or dosing)) or "gy radiation" or "radiation dose-response").mp. orchemoradiotherapy/ or chemoradiotherapy, adjuvant/ or Radiotherapy, Adjuvant/ or rt.fs. or radiotherapy/ or ((adjuvant adj2 chemotherap*) or chemoradiotherap* or radiochemotherap*).mp. | Population terms |
| 4 | 1 and 2 and 3 | FINAL |

**B) EMBASE**

| Set | History | Comments |
| --- | --- | --- |
| 1 | mucosa inflammation/ or stomatitis/ or aphthous stomatitis/ or aphthous ulcer/ or herpetic stomatitis/ or oral mucositis/ or (stomatitis or (oral adj5 mucositis) or (aphthous adj5 (stomatitides or ulcer*)) or (canker adj5 sore*) or aphthae or (simplex adj2 oral adj2 herpes) or ((herpet* adj5 gingivostomatitides) or gingivostomatitis) or (herpetic adj5 stomatitides)).ti,ab. or mouth mucosa/ or cheek mucosa/ or gingival/ | Oral mucositisterms |
| 2 | diode laser/ or phototherapy/ or low level laser therapy/ or light emitting diode/ or lasers/tu or ((laser* adj5 low adj5 (level* or power)) or "low-power laser*" or "low-level laser*" or (laser* adj5 (phototherap* or therap* or treatment* or treated or treating)) or lllt).ti,ab. or (lll or lle or "low-laser energy" or (low adj5 laser adj5 energy) or "light-emitting diode*" or (light adj5 emit* adj5 diode*) or LED or LEDs).ti,ab. | Laser therapy terms |
| 3 | exp neoplasm/ or exp antineoplastic agent/ or exp transplantation/ or exp radiotherapy/ or (neoplasm* or neoplas* or cancer* or oncolog* or tumor* or tumour* or transplant*).mp. or radiation response/ or radiotherapy/ or chemoradiotherapy/ or adjuvant chemoradiotherapy/ or radiation response/ or (((gray or sievert) adj2 unit*) or (radiation adj2 (dosage* or dose or dosing)) or "gy radiation" or "radiation dose-response").mp. or radiometry/ or radiation dose/ or rt.fs. or ((adjuvant adj2 chemotherap*) or chemoradiotherap* or radiochemotherap*).mp. | Population terms |
| 4 | 1 and 2 and 3 [****base clinical set****] | FINAL |

**C) EBM Reviews - Cochrane Central Register of Controlled Trials**

| Set | History | Comments |
| --- | --- | --- |
| 1 | mucositis/ or stomatitis/ or stomatitis, aphthous/ or stomatitis, herpetic/ or (stomatitis or (oral adj5 mucositis) or (aphthous adj5 (stomatitides or ulcer*)) or (canker adj5 sore*) or aphthae or (simplex adj2 oral adj2 herpes) or ((herpet* adj5 gingivostomatitides) or gingivostomatitis) or (herpetic adj5 stomatitides)).ti,ab. or Mouth Mucosa/ or ((oral or mouth) adj5 mucosa).ti,ab.mucosa inflammation/ or stomatitis/ or aphthous stomatitis/ or aphthous ulcer/ or herpetic stomatitis/ or oral mucositis/ or mouth mucosa/ or cheek mucosa/ or gingival/ | Oral mucositisterms |
| 2 | laser therapy/ or laser therapy, low-level/ or phototherapy/ or lasers/tu or ((laser* adj5 low adj5 (level* or power)) or "low-power laser*" or "low-level laser*" or (laser* adj5 (phototherap* or therap* or treatment* or treated or treating)) or lllt).ti,ab. or (lll or lle or "low-laser energy" or (low adj5 laser adj5 energy) or "light-emitting diode*" or (light adj5 emit* adj5 diode*) or LED or LEDs).ti,ab. or diode laser/ or phototherapy/ or low level laser therapy/ or light emitting diode/ | Laser therapy terms |
| 3 | exp neoplasms/ or exp Antineoplastic Agents/ or organ transplantation/ or exp tissue transplantation/ or transplantation, autologous/ or transplantation, heterologous/ or transplantation, heterotopic/ or exp transplantation, homologous/ or (neoplasm* or neoplas* or cancer* or oncolog* or tumor* or tumour* or transplant*).mp. or radiation dosage/ or dose-response relationship, radiation/ or Radiometry/ or Radiotherapy Dosage/ or (((gray or sievert) adj2 unit*) or (radiation adj2 (dosage* or dose or dosing)) or "gy radiation" or "radiation dose-response").mp. orchemoradiotherapy/ or chemoradiotherapy, adjuvant/ or Radiotherapy, Adjuvant/ or rt.fs. or radiotherapy/ or ((adjuvant adj2 chemotherap*) or chemoradiotherap* or radiochemotherap*).mp. or exp neoplasm/ or exp antineoplastic agent/ or exp transplantation/ or exp radiotherapy/ or radiation response/ or radiotherapy/ or adjuvant chemoradiotherapy/ or radiation response/ or radiation dose/ | Population terms |
| 4 | 1 and 2 and 3 | FINAL |
