# Supplementary material for: Effect of Prophylactic Low Level Laser Therapy on Oral Mucositis: A Systematic Review and Meta-Analysis
Source: PLoS One. 2014 Sep 8;9(9):e107418. doi: 10.1371/journal.pone.0107418 (PMC4157876; doi:10.1371/journal.pone.0107418)
Supplement: Appendix S2 — Risk of bias assessment for included studies*. (DOC) [file pone.0107418.s002.doc]

Supplemental Appendix 2. Risk of bias assessment for included studies*

| **Study ID** | **Random sequence generation** | **Allocation concealment** | **Blinding of participants and personnel** | **Blinding of outcome assessment** | **Incomplete outcome data** | **Selective outcome reporting** |
| --- | --- | --- | --- | --- | --- | --- |
| Antunes 2013 | Unclear | Unclear | Low risk | Low risk | Low risk | Low risk |
| Arbabi-Kalati 2013 | Low risk | Unclear | Low risk | Low risk | Low risk | Low risk |
| Gautam 2012 | Low risk | Low risk | Low risk | Low risk | Low risk | Low risk |
| Gautam 2012 | Low risk | Low risk | Low risk | Low risk | Low risk | Low risk |
| Gouvea de Lima 2012 | Low risk | Unclear | Low risk | Low risk | Low risk | Low risk |
| Hodgson 2012 | Low risk | Low risk | Low risk | Low risk | Low risk | Low risk |
| Hodgson 2012 | Low risk | Low risk | Low risk | Low risk | Low risk | Low risk |
| Oton-Leite 2012 | Low risk | Unclear | Low risk | High risk | Unclear | Unclear |
| Pires-Santos 2012 | Unclear | Unclear | Unclear | Unclear | Unclear | Unclear |
| Silva 2011 | Low risk | Unclear | High risk | Low risk | Low risk | Low risk |
| Chor 2010 | Low risk | High risk | Low risk | Low risk | Low risk | Unclear |
| Khouri 2009 | High risk | High risk | High risk | High risk | Low risk | Low risk |
| Antunes 2007 | Unclear | Unclear | Low risk | Low risk | Low risk | Low risk |
| Cruz 2007 | Low risk | High risk | High risk | Low risk | Low risk | Unclear |
| Schubert 2007 | Unclear | Unclear | Low risk | Low risk | High risk | Unclear |
| Lopes 2006 | High risk | High risk | High risk | High risk | High risk | Unclear |
| Arun Maiya 2006 | Low risk | Unclear | High risk | Low risk | Low risk | Low risk |
| Bensadoun1999 | Low risk | Unclear | Low risk | Low risk | Low risk | Low risk |
| Cowen 1997 | Low risk | Unclear | Low risk | Low risk | Low risk | Low risk |

* Risk of bias in each domain was classified as low risk, high risk or unclear risk of bias.
