# Supplementary figures and images for: Effect of Prophylactic Low Level Laser Therapy on Oral Mucositis: A Systematic Review and Meta-Analysis
Source: PLoS One. 2014 Sep 8;9(9):e107418. doi: 10.1371/journal.pone.0107418 (PMC4157876; doi:10.1371/journal.pone.0107418)

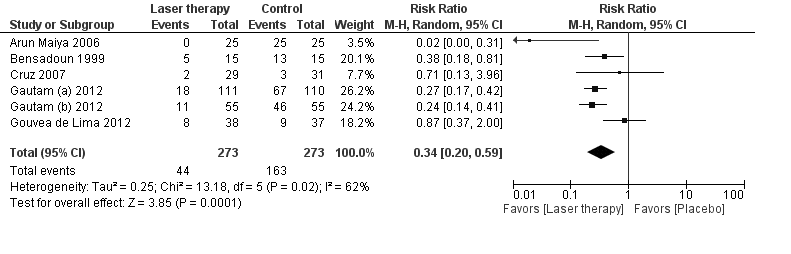

Supplement: Appendix S3 — Forest plot of incidence of severe (grade 3 or 4) mucositis at week 6±1 in head and neck cancer radiotherapy trials and at day 10±4 in chemotherapy or hematopoietic stem cell transplantation trials. Squares to the left of the vertical line indicate that low level laser therapy reduces mucositis. Horizontal lines through the squares represent confidence intervals (CIs). The size of the squares reflects each study's relative weight, and the diamond represents the aggregate risk ratio and 95% CI. (TIF) [file pone.0107418.s003.tif]
